# Antiviral Immunity in SARS-CoV-2 Infection: From Protective to Deleterious Responses

**DOI:** 10.3390/microorganisms9122578

**Published:** 2021-12-13

**Authors:** Grigore Mihaescu, Mariana Carmen Chifiriuc, Corneliu Ovidiu Vrancianu, Marian Constantin, Roxana Filip, Mihaela Roxana Popescu, Liliana Burlibasa, Anca Cecilia Nicoara, Alexandra Bolocan, Ciprian Iliescu, Gratiela Gradisteanu Pircalabioru

**Affiliations:** 1 Faculty of Biology, University of Bucharest, 030018 Bucharest, Romania; grigore.mihaescu@bio.unibuc.ro (G.M.); ovidiu.vrancianu@yahoo.com (C.O.V.); liliana.burlibasa@bio.unibuc.ro (L.B.); 2 Life, Environmental and Earth Sciences Division, Research Institute of the University of Bucharest, 050096 Bucharest, Romania; gratiela.gradisteanu@icub.unibuc.ro; 3 The Romanian Academy, 25 Calea Victoriei, Sector 1, 010071 Bucharest, Romania; 4 Institute of Biology, 060031 Bucharest, Romania; cvgmarian@gmail.com; 5 Faculty of Medicine and Biological Sciences, Stefan cel Mare University of Suceava, 720229 Suceava, Romania; roxana_filip@yahoo.com; 6 Regional County Emergency Hospital, 720284 Suceava, Romania; 7 Department of Cardiology, Elias Emergency University Hospital “Carol Davila”, University of Medicine and Pharmacy “Carol Davila”, 020021 Bucharest, Romania; roxana.popescu@umfcd.ro; 8 Faculty of Pharmacy, University of Medicine and Pharmacy “Carol Davila”, 020021 Bucharest, Romania; anca.nicoara@umfcd.ro; 9 General Surgery, University of Medicine and Pharmacy “Carol Davila”, 020021 Bucharest, Romania; bolocan.alexa@gmail.com; 10 National Institute for Research and Development in Microtechnologies—IMT, 077190 Bucharest, Romania; ciprian.iliescu@imt.ro; 11 Faculty of Applied Chemistry and Materials Science, University “Politehnica” of Bucharest, 011061 Bucharest, Romania; 12 Academy of Romanian Scientists, 010071 Bucharest, Romania

**Keywords:** immune response, antiviral, SARS-CoV-2, lymphocytes, antibodies, inflammation

## Abstract

After two previous episodes, in 2002 and 2012, when two highly pathogenic coronaviruses (SARS, MERS) with a zoonotic origin emerged in humans and caused fatal respiratory illness, we are today experiencing the COVID-19 pandemic produced by SARS-CoV-2. The main question of the year 2021 is if naturally- or artificially-acquired active immunity will be effective against the evolving SARS-CoV-2 variants. This review starts with the presentation of the two compartments of antiviral immunity—humoral and cellular, innate and adaptive—underlining how the involved cellular and molecular actors are intrinsically connected in the development of the immune response in SARS-CoV-2 infection. Then, the SARS-CoV-2 immunopathology, as well as the derived diagnosis and therapeutic approaches, will be discussed.

## 1. Introduction

Coronaviruses are a large group of enveloped viruses infecting both humans and a wide range of wild and domestic animals [1]. In 2002 and 2012, two highly pathogenic coronaviruses with a zoonotic origin emerged in humans and caused fatal respiratory illness, respectively, the severe acute respiratory syndrome coronavirus (SARS-CoV) and Middle East respiratory syndrome coronavirus (MERS-CoV). Now, we are experiencing the COVID-19 pandemic produced by SARS-CoV-2 [2,3]. The pattern of these infections enabled coronaviruses to be considered as emerging pathogens and a major public health concern for the twenty-first century [4,5,6]. Common features of coronaviruses are RNA genome with positive polarity, animal reservoir, broad host spectrum, and the potential for human-to-human transmission [4,5,6]. Bats are reservoirs for both epidemic coronaviruses and several other viruses with pandemic potential such as Ebola, Marburg, rabies [7,8].

Coronaviruses have a monostrand RNA genome, capped at the 5’ end, with a poly-A sequence at the 3’ end [7]. Two-thirds of the 5’ end is occupied by ORF1a/b and are translated into two polyproteins—1a and 1b, subsequently cleaved into 16 non-structural (NS) proteins: a chymotrypsin-like protease, a papain-like protease, helicase, and RNA-polymerase [9]. The other ORFs encode accessory proteins that interfere with the innate immune response [10,11]. A peculiarity, probably unique to RNA viruses, is the existence of the NS14 exoribonuclease domain with a proofreading role for the prevention of non-functional mutations [12]. The NSP2 and NSP3 play a vital role in the infectious potential of these viruses [13,14]. A number of 380 amino acid substitutions in NSP2, NSP3, protein S, and RBD were identified in SARS-CoV-2 and other SARS viruses [12,15]. SARS-CoV-2 has a nucleotide sequence identity of 96% with bat coronavirus and 79.5% with SARS-CoV [16]. Thus, SARS-CoV-2 has probably passed from animal intermediate to humans and spread with a high rate of infectivity in the unprotected population, reaching pandemic proportions in a very short time [17].

SARS-CoV-2 is the result of a recombination process [18], probably with a host cell mRNA, from which it acquired a 12-nucleotide sequence encoding the tetrabasic sequence Arg-Arg-Ala-Pro [19]. This sequence is the site of action of the furin-like enzyme that separates the two subunits of protein S, facilitating the fusion between virus and cell [20]. The presence of furin in the lungs, liver, and small bowel explains the tropism of the virus for these tissues, as well as the digestive symptoms observed in patients infected with SARS-CoV-2 (liver failure or diarrhea) [21,22]. Coronaviruses could also infect the intestinal tract, although it is not yet established whether the virus is resistant to gastric acidity, as well as to the detergent action of bile salts [23,24].

Risk factors for the evolution of SARS-CoV-2 infection in severe and critical forms are age, male gender, obesity, smoking, comorbidities (hypertension, type 2 diabetes, renal failure).

The question of the year 2021 is: will immunity, naturally or vaccine-induced, be effective against evolving SARS-CoV-2 variants, or will the variants be able to escape human immunity? In other words, will B.1.1.7, B.1.351, P.1, or other upcoming variants be able to escape naturally or artificially acquired active immunity as well as the effect of monoclonal antibodies [18].

This review aims to discuss the antiviral immune response in SARS-CoV-2 infection. Cellular and humoral immune response, as well as the SARS-CoV-2 immunopathology, will be discussed.

## 2. Antiviral Immune Response in SARS-CoV-2 Infection

Human viruses use different pathways to enter the host, such as respiratory (e.g., coronaviruses, influenza, adenoviruses, measles, varicella-zoster, and rubella viruses) or digestive tract (enteroviruses, hepatitis A virus, rotaviruses, noroviruses) [25,26], while others enter through skin lesions or fluids (blood, secretions) (e.g., HIV, hepatitis B and C viruses, herpes simplex viruses, arboviruses) [25,26,27]. At the entrance gates, the virus must overcome the first line of defense represented by the cutaneous—mucosal anti-infectious barrier, consisting mainly of physico-chemical mechanisms, the presence of normal microbiota, and the components of the subcutaneous and mucosa-associated lymphoid tissue (Table 1).

SARS-CoV-2, carried by saliva and nasal secretions released during speaking, shouting, sneezing, and coughing, infects the nasopharyngeal epithelium cells, having a 10–20 times higher affinity than other SARS viruses for the ACE2 receptor of respiratory tract epithelial cells. SARS-CoV-2 enters the cell through the mechanism of endocytosis or membrane fusion of peplos. The average incubation period before the onset of symptoms is variable, between 4 and 10 days, depending on the amount of infectious virus. However, symptoms appear after an average time of 11 days, during which the patient spreads the virus [28]. The disease is often asymptomatic. The clinical form has various symptoms, featuring mild, moderate, or severe cases. Most patients with severe disease develop ARDS (acute respiratory distress syndrome), accompanied by high rates of mortality, characterized by bilateral leukocyte infiltrate and hypoxemia (decreased arterial PO_2_/inspired PO_2_ ratio), requiring mechanical ventilation [29]. After entering epithelial cells, the virus, with an RNA genome of positive polarity, multiplies at a high rate. As a result, the viral load in the epithelium of the upper respiratory tract reaches its maximum value 5–6 days after the onset of symptoms. Clinical manifestations may differ from one individual to another, being influenced by insufficiently known genetic features. Nevertheless, the extent of the pathological process itself is primarily dependent on the reactivity of the innate and adaptive immune system components, which are activated and mutually potentiated in a complex network of cellular and molecular interactions. During the antiviral immune response (IR) triggered either by the viral infection and/or the vaccine administration, a complex interplay between the innate and adaptive immunity cellular and molecular components (Table 2) occurs, depending on the nature of the virus (structure, multiplication cycle, entry gate), as well as by the genetic predisposition of the host [30].

### 2.1. Antiviral Innate Response in SARS-CoV-2 Infection

The cellular effectors of the innate IR are represented by various phagocytic cells, DCs, and innate immune lymphoid cells, while the molecular effectors are represented by different antiviral molecules [31].

The innate immune phagocytic cells are in the first line of antiviral defense, being activated by pathogen- and danger-associated molecular patterns, as well as by metabolite-associated danger signals. The newly formed virions are detected by tissue macrophages as soon as they attach to the surface of epithelial cells. The macrophage activation is based on the detection of a wide variety of viral antigens through a limited number of pattern recognition receptors (PRR), expressed on macrophages, monocytes, dendritic cells (DCs), and neutrophils. PRRs are activated by DAMP (damage-associated molecular patterns) (dead cell contents, heat-shock proteins released after cell damage) and PAMP (pathogen-associated pattern). The best known PRR receptors are TLR (toll-like receptors) and NLR (nucleotide-binding oligomerization domain-like receptors) [32,33]. In the case of SARS-CoV-2, the early activation of the innate IR by specific PAMPs (viral RNA, oxidized phospholipids) seems to be essential to stopping the viral infection progression behind the entrance gate [34]. The SARS-CoV-2 infection triggers the activation of a cytoplasmic receptor (of the NOD-like family) in macrophages, epithelial, possibly even endothelial cells that contain the NLRP3 inflammasome. NLRs form support oligomers for caspase-1 activation. Active caspase-1 cleaves the family of pro-inflammatory cytokine interleukin (IL)-1 into the bioactive forms IL-1β and IL-18. In addition, TLR-3, -7, -8, and -9 receptors respond to viral RNA and activate the NF-κB pathway, followed by pro-inflammatory cytokine cascade activation [35]. Thus, activation of macrophages and release of neutrophil-attracting pro-inflammatory cytokines may limit the spread of the virus. The activation of the innate IR could either lead to viral infection clearance or to the activation of adaptive IR by the antigen-presenting cells (APC) [36]. The pro-inflammatory cytokines produced during the innate IR are tumor necrosis factor-alpha (TNF-α) and IL-1, which stimulate the expression of endothelial adhesion molecules, favoring the accumulation of neutrophils, macrophages, bloodborne dendritic cells, and NK cells to the site of infection [31]. Furthermore, the produced antiviral interferons (IFN) activate hundreds of interferon-stimulated genes (ISGs), their products exhibiting antiviral effects, such as viral nucleic acid degradation and inhibition of viral gene expression [31].

IFN I (α, β) synthesis represents the primary, essential form of a protective response to SARS-CoV-2 infection. IFN is a powerful molecular messenger whose synthesis is induced after the detection of intracellular PAMP viral components (e.g., viral RNA) by the RIG-I (retinoic acid-inducible interferon gene) and MDA5 (melanoma differentiation-associated gene 5) recognition sensors of the innate immunity cells (monocytes/macrophages, DC, neutrophils).

NLR and RLH (rig-like helicases) are soluble cytoplasmic proteins that monitor the intracellular environment for intracellular invaders (PAMP) and DAMP signals. After stimulation of the two helicases, NF-κB and IRF3/7 are activated, and gene transcription for IFN I is induced [32,34,37]. IFN I binds to specific membrane receptors (IFNAR) and triggers the expression of hundreds of ISGs, with different functions, including direct inhibition of viral multiplication, NK cell recruitment, and activation of adaptive immune response cells. Rapid and robust synthesis of IFN limits the multiplication of SARS-CoV-2, and the infection is either asymptomatic or has a mild form. IFN III (λ) is restricted to the mucosal surface, and it is assigned with a protective role, as it does not induce inflammation [38]. SARS-CoV and seasonal coronaviruses (229E, NL63, OC43, and HKU1) induce a weak IFN synthesis compared to other RNA viruses. SARS-CoV- suppresses IFN release in vitro and in vivo, and SARS-CoV-2 has the same effect, suggested by the absence of IFN I and IFN III (λ) in infected cell lines, in primary bronchial cell cultures, and in vivo, (on ferrets). The serum concentration of IFN I is undetectable by conventional methods, although ISGs are expressed, suggesting that a small amount of IFN I is required for their activation. The ability of different tissue cells to respond to IFN I differs depending on their physiological state. The plasmacytoid DC (pDC) produce 1000 times more IFN I than any other cell type and are resistant to the multiplication of most viruses [39].

An important part of the innate antiviral immunity is represented by the intrinsic immunity, which is mediated by constitutively expressed or induced factors that recognize specific viral components and restrict the viral multiplication cycle, rendering host cells non-permissive to a certain virus [40]. These proteins could be included in the PRR category, as they bind directly to viral components; however, unlike other PRRs that inhibit viral infection indirectly by inducing interferons and other antiviral molecules, intrinsic antiviral factors block the viral replication immediately and directly, often before the onset of the IFN response [41]. The expression of intrinsic antiviral factors in different cells and individuals dictates the permissiveness or non-permissiveness of a cell type to a certain virus. They also play an important role in limiting cross-species transmission of a virus and thereby determining the viral tropism [41].

### 2.2. Antiviral Adaptive Immune Response in SARS-CoV-2 Infection

The cells of the adaptive immunity are B and T cells, while the adaptive humoral immunity is mediated by antibodies. During the adaptive antiviral IR, both cell-mediated immunity (CMI) and humoral-mediated immunity (HMI) are important for resolving viral infection [42]. CMI eliminates the infected cells, thus stopping the viral multiplication cycle. Meanwhile, the main effectors of the specific HMI neutralize the free viral particles present in the human body and recognize the viral antigens exposed by the infected cells, thus activating the antibody-dependent cell-mediated cytotoxicity (ADCC) or complement activation [31].

#### 2.2.1. Humoral-Mediated Immunity-Activation of B Cells

Some viral antigens are T-dependent so that, for the HMI activation, B cells require cooperation with Th2 cells [43]. Ig receptors of specific B cell clones recognize the native spatial conformation of virion surface antigens (Ag) or soluble viral Ag circulating in the internal environment [44]. B cell-embedded Ag is processed in the cytoplasm and presented in association with membrane major histocompatibility complex (MHC) II molecules, where CD4+ T cells specifically recognize them. Depending on the set of released cytokines, CD4+ T cells are classified into two subpopulations: Th1 and Th2 [45]. Th1 cells release CMI-stimulating IL-2 and IFNγ, mediating the differentiation of CD4+ and CD8+ T cells, which are dominant in viral or intracellular bacterial infections [46]. The Th2-type cytokines IL-4, -5, -13 stimulate B cell proliferation and differentiation, which has a dual role, being both APC for Th2 cells and HMI effector cells. Activated clones proliferate and synthesize specific IgM, IgA, and IgG [38,47].

In the acute stage of the infection, the specific Ab titer is barely detectable but reaches its maximum value at 2–4 weeks and persists for weeks, months, or even the entire life (e.g., for yellow fever and measles virus), playing an essential role in preventing reinfection and newborn protection [48]. Specific Ab interact with extracellular, structural, or non-structural viral Ag, the main effect being the neutralization of viral particles, i.e., the loss of virion infectivity [49]. The binding of Ab to viral Ag exposed on the infected cell’s surface can block the release of virions. Activation of complement cascade or of ADCC causes lysis of the infected cell (cytolysis) [50].

However, non-cytolytic Ab can mask viral Ag exposed to the cell surface, which is inaccessible to lytic effector cells (CD8+ cytotoxic T cells, NK cells) [46]. Ab have an essential contribution in ending the viremic phase of the infectious process by reducing viremia, decreasing infectivity, reducing the number of infected cells, and the burden on CD8+ cytotoxic T cells [51]. Long-term maintenance of elevated serum Ab titers requires continuous synthesis of these molecules, even in the absence of Ag by the memory B cells [52]. If the infection is persistent, viral Ag will recruit new B cells [53]. During the secondary infection, antibodies are synthesized rapidly, at a higher titer [54].

Antiviral Ab synthesized during the primary immune response (PIR) have multiple specificities, low binding energy (affinity), and thus, easily dissociate; therefore, residual infectivity could persist. In some cases, Ab do not cover the critical sites of the virions and does not neutralize the viral particles [55]. Neutralizing Ab are synthesized after affinity maturation and are essential for protection against reinfection with the homologous virus [56].

Sometimes, Ab stimulate viral infectivity when the virion-Ab immune complexes, through the Fc region of Ig, bind to the Fc receptor of monocytes/macrophages and granulocytes and stimulate viral particle internalization [57]. This phenomenon, called ADE (antibody-dependent enhancement), has been demonstrated in vitro for Bunya, Corona, Flavi, Ortho, Paramyxo, Retra, and Rhabdo and Togaviruses [58,59,60].

The standard test for detecting neutralizing Ab in the case of SARS-CoV-2 infection is using pseudovirions with liposome-associated S protein instead of virions. Other methods are ELISA and the lateral flow assay (LFA), in which the Ag is represented either by the integral S protein, the S1 fragment, or RBD [61,62]. A positive serological test indicates previous exposure to one or more viral epitopes, but false-positive results could occur if the pathogen or even the vaccine share common epitopes with other infectious agents. However, Ab detection is essentially helpful for the early diagnosis of infection, for the diagnosis of suspected infection cases with negative RT-PCR test, and for identifying asymptomatic infections [63]. The seroconversion for total Ab, IgM, and IgG was 11, 12, and 4 days, respectively. In the serum of uninfected children and adolescents, anti-S1 and S2 specific IgG, along with IgM and IgA, were detected as proof of an earlier seasonal coronavirus infection and their cross-reaction to the S protein of SARS-CoV-2 [64]. In SARS-CoV-2 infection, Ab specific for protein S and N are detected, on average, 10–14 days after the onset of symptoms. A longitudinal study of Ab dynamics over a 3-month interval after the onset of symptoms in 113 hospitalized patients revealed that IgM synthesis precedes IgG synthesis, reaching a maximum titer within 2–5 weeks, and decreases in 3–7 weeks after the symptoms’ disappearance. IgG reaches maximum titer in weeks 3–7 post-infection, and the plateau persists for at least 8 weeks. The neutralizing Ab are detected at 7–15 days, reach a maximum at 14–22 days, then decrease. The titer is lower in asymptomatic patients or those with mild forms of the disease. Cross-reactivity with other coronaviruses is limited [61,65]. Specific anti-RBD Ab are essential for the protective, neutralizing effect, while anti-non-RBD Ab may have an ADE effect [66]. Anti-SARS-CoV-2 neutralizing Ab are 90% anti-RBD and anti-N protein and correlated with the clinical scores of the 647 subjects analyzed by a high-resolution serological method. Anti-RBD Ab have a rate of 1/2 at 49 days, but in some patients, the titer increases due to increased maturation affinity [67]. Assessing the duration of protective immunity is essential for understanding vaccination, plasma therapy, or monoclonal Ab treatments [62]. Convalescent subjects have high plasma levels of anti-RBD, anti-S2, and anti-N IgG [68]. However, after an average of 39 days from the onset of symptoms, specific anti-RBD Ab titers in convalescent patients decrease. Specific anti-RBD IgG and IgA decrease significantly 6–10 weeks after the onset of symptoms, and IgM titer has a much faster decrease [69].

The amplitude of HMI was correlated with the severity of the pathology. Ab at a neutralizing titer are synthesized after at least 8 days from the onset of symptoms in 95% of patients. After 60 days, the titer decreases with individual variations: after 94 days, some have a neutralizing titer, but in most cases, the titer decreases to undetectable values, a common feature of seasonal coronavirus infections [70]. The practical conclusion is that in order to be effective in treating SARS-CoV-2 infection, plasma must be harvested shortly after the disappearance of symptoms [71].

Anti- SARS-CoV-2 Ab were also detected in saliva. A longitudinal study of serum (439 samples) and saliva (128 samples) Ab titers over 3–115 days after symptoms, compared with negative control samples, showed that the maximum titer of anti-RBD Ab is reached in an interval of 16–30 days in both fluids. In saliva, IgA decreases rapidly, while in serum, IgM and especially IgG levels are maintained for at least 3 months in most samples. Therefore, measurements of IgG in saliva may indicate specific immunity, showing a correlation with serum IgG titer [72].

People with asymptomatic infections activate a low-intensity IR and have lower levels of pro-inflammatory cytokines. As a result, they produce viruses for a more extended period than the symptomatic ones. Furthermore, they become negative for IgG to a greater extent than symptomatic ones immediately after infection [70]. Convalescent subjects after SARS-CoV-2 infection have high titers of anti-protein S (RBD and S2) and anti-N IgG and large populations of reactive memory B cells [68].

Depending on the reactivity of the HMI, a proportion of the infected individuals remain carriers of the virus for a variable interval. The incubation period of SARS-CoV-2 is 1–14 days, with an average of 5–6 days. Viral RNA levels decrease rapidly in the second week of the disease and may become undetectable in the nasopharynx by RT-PCR assay [73]. After meeting the quarantine discontinuation conditions with the negative nasopharyngeal/oropharyngeal RT-PCR test, some patients returned positive to the RT-PCR test after 5–13 days [74]. The severity of the pathology does not correlate with the duration of persistence. Persistent infection is a feature of coronaviruses, probably caused by the fact that the virus envelops in intracellular membrane structures (endoplasmic reticulum, Golgi cisterns), and epithelial cells are ineffective in presenting viral Ag to the membrane. Carmo et al., (2020) identified patients with mild disease who remained positive, persistently infected for a longer interval (51 days until the first negative test) than those with severe disease [75]. The new genomic variants may cause reinfection, with severe symptoms [76,77,78]. The increase in the level of infectivity of the new strains is the result of a process of adaptation of SARS-CoV-2 to the human host, which probably consists of the ability to multiply in a shorter cycle, to initiate the infectious process with a smaller number of virions, to attach more firmly to the ACE2 cell receptor, or even to infect a broader spectrum of cells. *Macacus rhesus* infection with SARS-CoV-2 by tracheal application produced a moderate infection with interstitial pneumonia and high titer viral excretion. Reinfection with the identical viral strain in the early recovery phase from the initial infection did not produce detectable viral dissemination or clinical manifestations. However, the HMI response after secondary infection revealed a higher titer of neutralizing Ab [79].

#### 2.2.2. Cell-Mediated Immunity-Activation of T Cells

**Figure 1:**
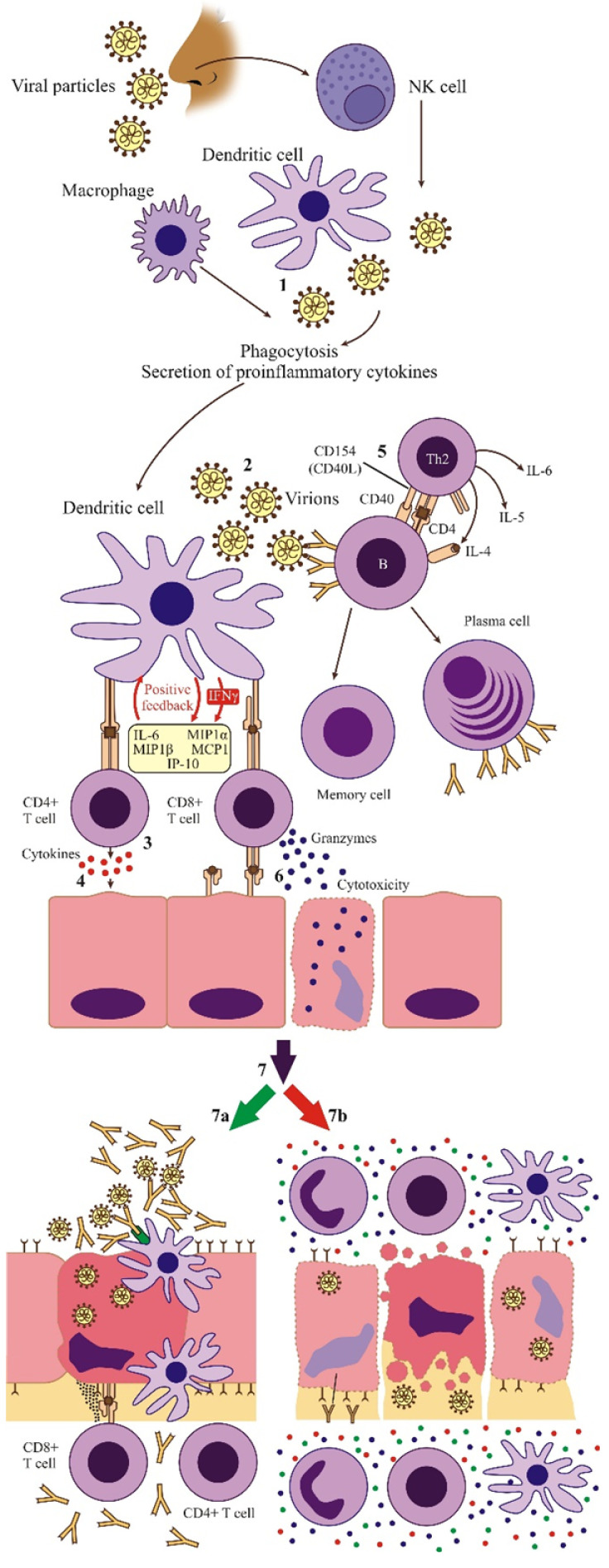
The evolution of the IR in SARS-CoV-2 infection. The virion (1) is recognized by innate immunity cells (1) and by the Ag receptors of Ag presenting cells (e.g., dendritic cells) (2,3) and B cells (4). After internalization and processing of virions, viral antigenic peptides are exposed on the surface of Ag, presenting cells in association with MHC I and II molecules (3). Activated CD4+ T cells release cytokines (TNFα, IL-2, IFNγ) that activate CD8+ cells, leading to proliferation and differentiation in effector cells (4). The T cells release cytokines (IL-4, 5, 6) with a regulatory function of the specific response of B lymphocytes to viral Ag (5). B cells respond by proliferation and generation of memory and plasma cells. Activated CD8+ T cells disseminate into the host tissues and subsequently exert cytotoxic effects on infected cells (6). In individuals with normal immunoreactivity, the initial inflammation triggered by infection of the epithelium attracts T cells and macrophages that induce the infected cells before the release of progenitor virions and neutralizing Abs block the spread of virions. Hence, the infectious process is stopped with minimal tissue damage and subsequent recovery (7a). In the case of an exaggerated inflammatory response, accumulation of inflammatory cells in the infected tissue results in overproduction of pro-inflammatory cytokines, which may lead to multiorgan damage (7b) (adapted after Tay et al., 2020 [96]).

Cell-mediated immunity (CMI) is the principal mechanism of antiviral specific defense, preceding Ab synthesis in all viral infections, but especially in cytolytic ones [80]. T cells recognize viral Ag only after processing and presentation by APC in association with MHC molecules (Figure 1). Among APC, the bridge between the innate and adaptive antiviral IR is accomplished by DCs, which are generated in the bone marrow, from the lymphoid, i.e., plasmacytoid DC (pDC) and myeloid (mDC) lines. They are well represented at the host entry sites and are not prone to infection. Furthermore, their response is not inhibited by viral proteins, which facilitates their function as sensors of infection [39,81,82]. In the early stage of viral infection, interstitial DCs (IDC) take up viral Ag at the virus gateway by phagocytosis, receptor-mediated endocytosis, or pinocytosis, and migrate to the regional lymph nodes and spleen. DCs present the Ag in association with MHC I and MHC II molecules to both naive CD8+ and CD4+ T cells [83]. Any protein encoded by the virus, structural or NS, can be processed by infected cells or by accessory cells of the immune response. The CD4+/CD8+ T cells indirectly recognize, through the membrane T cell receptor (TCR), viral Ag processed as short peptides (8–12 amino acids) and presented on the surface of infected cells in association with MHC II/MHC I molecules [84]. From the secondary lymphoid organs where they were activated, CD8+ and CD4+ T cells disperse in the body and accumulate at the site of infection. CMI reaches its maximum intensity two days after infection and mediates the early lysis of the infected cells at the end of the virus multiplication cycle [80].

CD4+ T cells synthesize cytokines that stimulate and attract macrophages, neutrophils, and essential antiviral effector cytokines with nonspecific actions (IFNγ), responsible for inducing the antiviral state of sensitive cells and exhibiting a cytotoxic effect. Activated CD4+ T cells also stimulate the differentiation of B cells and Ab synthesis. The CD8+ T cells produce a cytotoxic (lytic) effect on infected cells, mediated primarily by granzymes and perforin. In addition to their direct cytotoxic action, CD8+ T cells also produce antiviral effector cytokines (IFNγ, TNFα) and chemokines [50]. Activation and proliferation of CD4+ and CD8+ T cells, and an increased count of activated circulating B cells (plasmablasts), indicate the building of an effective antiviral IR [81].

In most patients infected with SARS-CoV-2, T cells are activated by epitopes located mainly on proteins S, M, and N. The total number of lymphocytes and those with anti-S specificity correlates with the anti-S Ab titer [85]. Peng Y et al., (2020) identified 41 peptides containing epitopes recognized by CD4+ and/or CD8+ T cells, correlated with anti-protein S Ab synthesis. Activation of CD+ T cells in the acute phase, and convalescents, has been associated with less severe disease. It was observed that in the absence of detectable neutralizing Ab, the patients with CMI have been cured without hospitalization, while those with neutralizing Ab, but without detectable CD4+ and CD8+ T cells underwent a fatal evolution [86]. T cells activated by seasonal coronaviruses cross-react with SARS-CoV-2, due to the high degree of identity of the amino acid sequence of S peptides. Cross-reactivity may explain different clinical manifestations of infection, from asymptomatic to life-threatening [87].

The immune response in SARS-CoV-2 infection is influenced by several factors, such as the HLA haplotype (different ability of MHC molecules to present the viral epitopes to T cells), dose of infectious virus, presence of immunodeficiency, IFN I level, immunotype of adaptive CMI, and onset of an adequate innate IR [66,88].

The different reactivity and clinical manifestations of SARS-CoV-2 infection in children and young people, as compared to adults, could be explained by the particular aspects of the IR in different age groups [89]. SARS-CoV-2 infection in children and young people is often inapparent, and the clinical one is mild or moderate. The mortality rate is lower compared to adults, even in those with multisystemic inflammatory syndrome. In adults, the neutralizing Ab titer is higher, as well as the pro-inflammatory IL-17A and IFNγ concentrations [90]. The increase in the proportion of Th17 is associated with the chronic evolution and progression of inflammatory processes similar to those found in some intestinal inflammatory diseases. The superior resistance of children and adolescents to SARS-CoV-2 infection is also thought to be due to their recent infections with seasonal coronavirus infections providing them with cross-protective immunity. However, infection with seasonal coronaviruses is not associated with humoral or cellular immunological memory, but pre-existing IgG, specific to the S1 subunit containing the RBD sequence is detected in uninfected children and adolescents. Seasonal coronavirus infection could induce anti-S1 and anti-S2 IgG, IgM, and IgA synthesis as a cross-humoral response to coronavirus protein S [64,91]. In the same age groups, 20–50% of the population has anti-SARS-CoV-2 T-reactive cells without exposure to the virus. However, after exposure to SARS-CoV-2, these T pre-activated cells do not respond intensely [92,93]. Anti-SARS-CoV-1 HMI appears to be long-lasting. Twenty-three individuals who underwent SARS-CoV-1 infection in 2003 have robust immune memory mediated by CD4+ T cells, which cross-react with SARS-CoV-2 S, M, N proteins, but do not cross-react with MERS homologous proteins [94,95].

### 2.3. Deleterious Effects of the Immune Response in SARS-CoV-2 Infection

The critical point of the evolution of SARS-CoV-2 -associated pathology is the transition from a protective immunity to an exacerbated inflammatory response and a significant decrease of the adaptive immune reactivity. The components and evolution of the IR in SARS-CoV-2 infection are presented in Figure 1.

Deficiency of IFN I and II synthesis has been correlated with severe forms of disease in the case of SARS-CoV-2 infection [97,98]. From the 27 proteins: 4 structural (S, E, M, N), 16 NS, and nine accessory proteins encoded by SARS-CoV-2, and at least 10 NS proteins allow the virus to escape or counteract the IFN I antiviral status induction. In addition, some NS proteins interact with cellular mRNA and non-coding RNA involved in protein synthesis. For example, NSP16 suppresses cellular mRNA synthesis, and NSP8 and NSP9 interfere with protein trafficking to the cytoplasmic membrane. Disruption of these functions suppresses the cell’s IFN I response pathway to infection [99,100]. Moreover, IFN I could also exhibit pathogenic effects, as a small set of ISG genes has proviral activity by stimulating ACE2 expression on lung pneumocytes, enterocytes, and mucus-secreting cells of the nasal mucosa [101]. Double-stranded RNA-dependent protein kinase (PKR-eukaryotic protein synthesis initiation kinase-eIF-2), which inhibits viral mRNA translation, is encoded by a gene in the ISG set. A MERS protein blocks PKR activity, but SARS-CoV-2 interference with this protein is unknown [98].

Delayed synthesis of IFN I, due to a genetic or physiological deficiency, allows viral multiplication before ISG expression. Thus, the SARS-CoV-2 infection progresses to the clinical phase. In contrast to those with mild and moderate pathology, patients with severe and critical forms show a diminished response to IFN I, with undetectable IFNβ and low IFNα levels, a condition that precedes clinical deterioration. In patients with severe forms of the disease, pDCs have a diminished ability to produce IFNα and TNFα [102].

In the absence of IFN as an antiviral mechanism, the body activates an inflammatory response, which, especially in the lungs, has a pathogenic role: pulmonary edema, accumulation of monocytes/macrophages in the lung parenchyma, and secretion of pro-inflammatory cytokines (IL-1, IL-6) and chemokines (IP-10 = IFN induced protein 10, MIP1α, MIP1β, MIP-1 = monocyte attractant protein 1) attractive to monocytes, macrophages, and T lymphocytes at the site of infection. The monocytes and macrophages accumulated in the lung parenchyma synthesize an excessive amount of pro-inflammatory cytokines, producing a systemic cytokine storm, responsible for multiorgan lesions [103,104]. The persistence of activated macrophages and neutrophils amplifies the release of IL-1β and IL-6 and the recruitment of neutrophils, monocytes, and lymphocytes. In the pulmonary parenchyma, activated neutrophils release leukotrienes which attract neutrophils. The inflammatory flood compromises the interstitial tissue and alveoli [105]. In severe cases, due to impaired immune function, SARS-CoV-2 infects type II (secretory) pneumocytes and CD169+ macrophages (both expressing ACE2) but does not infect T or B lymphocytes. Cytokines released by infected lung cells are attractive to T lymphocytes in the parenchyma, which synthesize IFNγ and amplify the inflammatory response, associated with edema and extensive clinical manifestations, such as severe respiratory distress syndrome (ARDS), 8–9 days after the onset of symptoms [61,106,107].

Many therapeutic approaches in COVID-19 include the use of immunosuppressive or immunomodulatory drugs, as well as of monoclonal antibodies targeting pro-inflammatory effectors or contributing to reestablishing the protective immunity (Table 3).

Plasma levels of IL-6 increase in all chronic inflammatory reactions but also in the cytokine storm associated with SARS-CoV-2 infection [128,143]. It is well known that the pathogenesis of SARS-CoV-2 is amplified by pre-existing comorbidities of the cardiovascular (hypertension, diabetes) and renal systems, as well as by obesity, which decrease the body’s ability to adapt and tolerate high systemic cytokine concentrations. Viremia confirmed by RT-PCR analysis is associated in critically ill patients with a 10-fold increase in the concentration of IL-6 [105]. The elevated serum level of IL-6 is a marker of the need for mechanical ventilation (Table 4) and reflects its role in recruiting other mediators of the inflammatory reaction [144]. Therefore, measurement of the most critical concentration (IL-1, IL-2, IL-6, TNFα), markers of the amplitude of the inflammatory process, is necessary for the prognosis of the pathology and the identification of antagonists as a therapeutic measure.

In severely or critically ill patients, the increased levels of serum cytokines (CRP, IL-1, IL-2, IL-6, TNFα, GM-CSF, IFNγ, NF-kB, IP-10, MCP-1, MIP-1a, MIP-1b) and lactate dehydrogenase (LDH) promote systemic thrombosis (disseminated intravascular coagulation—CID), haemophagocytosis, lymphohistiocytosis, and multiorgan dysfunction, a typical feature of the septic condition. Transcriptomic analysis of the broncho-alveolar lavage fluid of patients with mild disease revealed an increase in the expression of IL-1, IFNγ, IP-10, MCP-1, while in critical patients, it showed an increase in the expression of receptors for GM-CSF chemokines, IP-10, MCP-1, MIP-1a, and MIP-1b, indicators of increased activating signals that direct the migration of immune cells to the site of infection [149]. Pyroptosis releases cytoplasmic components that function as DAMPs and amplifies the release of pro-inflammatory cytokines.

**Figure 2:**
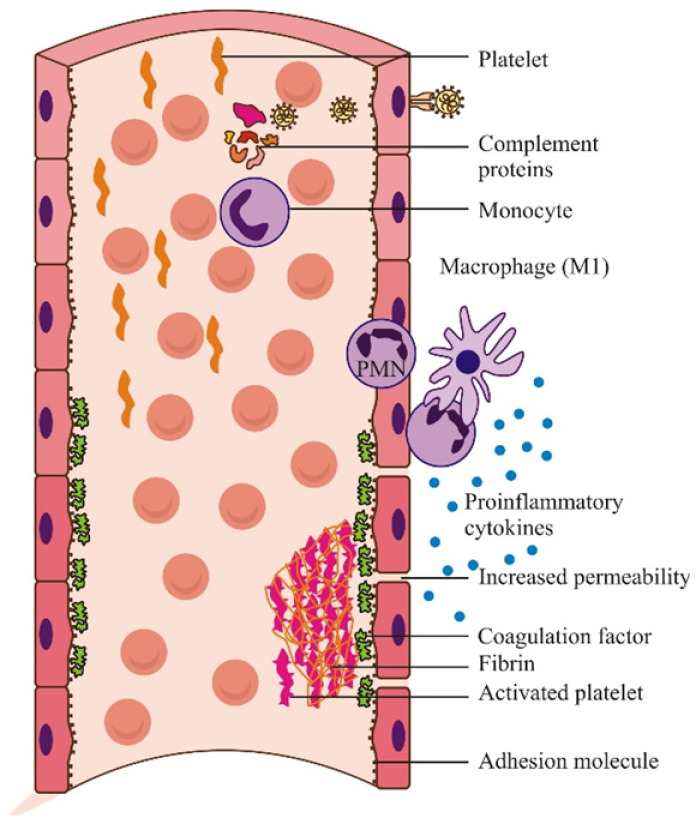
The interrelation between inflammatory and coagulation cascades in the SARS-CoV-2 infectious process. The inflammatory response increases vascular permeability and activates the complement macrophages and procoagulation phenotypes of platelets and endothelial cells, leading to endothelial lesions (reconstructed after Aid et al., 2020 [152]).

Cytokine storms produce multiple pathophysiological effects and are clinically manifested by ARDS and CID. GM-CSF stimulates medullary granulopoiesis and the circulation of neutrophils and monocytes, which synthesize pro-inflammatory interleukins. Several cytokines induce the febrile state and cell death, especially of the endothelium of the pulmonary and renal vessels. Cell lysis increases the permeability of blood vessels, causes edema, and lowers blood pressure. A consequence of the major pathological significance of the cytokine storm is CID, a reflection of the interaction of cytokines with hemostasis (discontinuity of the endothelium due to lysis causes platelet aggregation and the formation of white platelet thrombi). Damaged endothelial cells release the tissular factor (thromboplastin), the initiator of erythrocyte adhesion, and the formation of resistant red thrombus. Cytokines induce the excessive activation of complement, which causes thrombus formation [150] (Figure 2). In experimental infection in mice, Clay et al., (2020) found that the maximum concentration of pro-inflammatory cytokines was reached after the multiplication of the virus in the lung tissue was stopped [151]. Aid et al., (2020) highlighted vascular endothelial rupture, thrombosis, and cytokine storm markers on histopathological sections in human lung tissue and rhesus [152]. The virus passes into the blood, probably infecting and lysing endothelial cells, which determines the formation of multiorgan thrombi, with a lethal effect [153].

Anti-IFNα2 and anti-IFNωI auto-Ab were detected in 14% of patients with severe disease but not in those with mild or asymptomatic infections. Anti-IFN I auto-Ab are not induced by infection; they pre-exist and are responsible for 37% of deaths and 94% acute pneumonia in men, suggesting that the risk gene for inducing auto-Ab synthesis is located on the X chromosome [154].

In 74% of patients with severe and critical forms, three regions of chromosome three were identified as being directly or indirectly involved in pro-inflammatory pathology, being associated with IFN dysfunction: IFNAR2 gene, OAS, DPP9 (dipeptidyl peptidase 9), and TYK2 genes (tyrosine kinase 2). The IFNAR2 gene encodes the IFNα receptor. Gene mutations are rare, but tissue cells do not respond to the activating signal of viral multiplication inhibitory protein synthesis in the absence of receptor functionality, which explains 4% of severe cases. OAS (oligo-adenosine synthetase) genes encode proteins that activate the enzyme that degrades viral RNA. Mutation of a gene in the OAS group blocks the activation of the enzyme, and the virus multiplies. The DPP9, TYK2 genes amplify the inflammatory response triggered by the virus [155].

Overactivation of the NLRP3 inflammasome appears to play a role in initiating and maintaining the inflammatory state, as also demonstrated for metabolic and neurodegenerative diseases [156]. This mechanism seems to be involved in the SARS-CoV-2 immunopathological response. In bats, which tolerate SARS-CoV-2 infection, following an adaptive interaction of tens of millions of years, IFNα synthesis is constitutive. However, the activation of extensive inflammation is prevented by an anti-inflammatory mechanism, consisting of the suppression of the NLRP3 inflammasome, a cytoplasmic sensor of innate IR cells, recognizing PAMP and DAMP. Consequently, macrophages produce mainly the anti-inflammatory IL-10 [157].

ACE2, the SARS-CoV-2 receptor, an Ang II-degrading carboxypeptidase, is well expressed on the surface of mucosal and mucociliary cells of the nasal and bronchial epithelium, on the enterocytes of the ileum, and the cells of the corneal epithelium. It also appears to be expressed in the myocardium [158] and vascular smooth muscle cells [159], with an increased expression in diseased, compared to non-diseased, hearts [160,161].

The expression of ACE2 on the surface of the cells is correlated with the initial symptoms and late organ damage. It is co-expressed with the TMPRSS2 co-receptor (serine protease S2 transmembrane). Another co-receptor is DPP4 (dipeptidyl peptidase 4) [162]. However, in many tissues, especially fetal, the two molecules are not co-expressed [163]. After binding to the virus, ACE2 is cleaved by a metalloprotease (ADAM 17—a disintegrin and metalloprotease domain 17) and passes into the fluid of the epithelial surface. Activation of ADAM 17 modifies the epithelial receptor of IL-6 (IL-6R) to the soluble form, which induces a cascade of pro-inflammatory events. IL-6R is a functional marker of cellular senescence, and its serum levels growth is associated with an increased risk of severe evolution of COVID-19 disease in the elderly [88]. The virus downregulates ACE2, thus altering its anti-inflammatory role [159]. In patients with severe and critical forms of infection, decreased ACE2 function amplifies the effects of Ang II, with increased vascular permeability and inflammatory reaction, which causes acute lung damage due to RAS (renin-angiotensin system) dysfunction. There was initial concern regarding whether the RAAS blockade used in hypertensive patients might be deleterious in the context of SARS CoV-2 infection by raising the expression of ACE2. However, these drugs do not increase the expression of membrane-bound ACE2 [158] and do not cause greater myocarditis susceptibility. The serological picture of the response is dominated by the increased concentration of pro-inflammatory cytokines [163]. Viral infection can cause pyroptosis, a highly inflammatory form of primary ciliary dyskinesia (PCD), common to viruses that cause PCD.

Cardiovascular disease is known to be a major risk factor for unfavorable outcomes in SARS-COV-2 infection, which could also cause myocarditis, pericarditis, heart failure, arrhythmia, etc. [159]. As ACE2 is downregulated in COVID-19, the unopposed effects of angiotensin may cause heart failure, hypertension, thrombosis, and inflammation [164]. Quite a large proportion of COVID-19 patients (8–28%) have myocardial injury and elevated troponin levels. Moreover, troponin and NT pro-BNP (N terminal pro-brain natriuretic peptide) release have prognostic value in this category of patients [159].

Although the causes of the higher mortality rate of COVID-19 in men are hypothetical, some of the reasons seem to be related to different alleles of ACE2, which is encoded by an X chromosome gene, and to the immunoregulatory functions of sex hormones (estrogen and testosterone) [163]. The necropsy of male patients showed the absence of GC in the ganglia of the thoracic duct and the spleen, with a severe decrease in Bcl-6+ B and T cells, increasing the number of Th1 lymphocytes and aberrant TNFα synthesis in the same structures. In addition, specific SARS-CoV-2 B cells accumulate in the blood [165]. Furthermore, men have higher levels of IL-8, IL-18, and circulating monocytes, while women have a more intense T cell response. Severe disease progression in women has been correlated with high levels of plasma cytokines [166].

In comparison with asymptomatic contacts, harboring high counts of NK cells and an early and transient increase of IgA and IgM, and less of IgG, patients with severe infection have shown a marked increase in monocytes, high levels of IgA, and persistent IgG, synthesized relatively late in comparison with patients with a mild infection, in which a moderate monocyte growth and different dynamics of Ab synthesis were reported [144]. In patients with mild disease confirmed by RT-PCR, specific Ab titer decreases rapidly after 90 days [167]. In patients with severe disease, serum IgA occurs two days after symptoms and has a higher titer than in those with mild disease, being associated with pulmonary embolism and kidney damage [168,169]. The sIgA synthesized in mucosal lymphoid structures have a protective role, but at the same time, sIgA could induce the synthesis of IL-6, IL-8, MIP-1 (monocyte attractant protein 1), and GM-CSF [169].

Lymphopenia (800 T cells/μL) induced by SARS-CoV-2 is a constant in all patients with severe pathology. Histopathologic analysis of dead patients’ lymph nodes tissue samples has shown a lack of germinal centers (GC), a substantial decrease in B lymphocytes in GC, parallel to the abundance of Th1 cells, and aberrant production of TNF-α in the lymph nodes. CG loss was accompanied by extrafollicular activation of B lymphocytes. This could explain the marked lymphopenia of SARS-CoV-2 patients, absence of IgM to IgG class switching, and low levels of neutralizing serum Ab, which remained decreased even in late convalescence in some patients. Th extensive extrafollicular activation of B lymphocytes was not accompanied by memory or affinity maturation of B cell receptors [170]. Another cause of lymphopenia is that CD function is impaired and blocking their migration from the infectious focus to secondary lymphoid organs, leading to decreased activation of T cells [62,88,171,172,173]. Eosinophils are effector cells of nonspecific antiviral immunity, but they are also APC and have a role in building a specific IR to some respiratory viruses. Their number decreases by half and correlates with lymphopenia [35]. In patients with severe disease, the number of uncommitted T lymphocytes increases, the proportion of polyfunctional memory and Tregs that regulate immune reactivity decreases, and the proportion of reactive Th 17 and CD8+ cytotoxic T cells increases [35,36,174]. The activation of CD4+ T cells has been shown to be much more intense in patients with severe disease [36,175]. Thus, immunotype identification can individualize therapeutic immunological interventions. Three immunotypes of patients with severe forms of infection have been identified: (i) with robust activation of CD4+ T cell, (ii) with an intense CD8+ T cells response and weaker CD4+ T and B cells, and (iii) low percentage of activated lymphocytes (20%). The coordinated functionality of the three components of adaptive immunity—respectively, CD4+, CD8+, T cells, and Ab synthesis—are associated with milder disease. In individuals over 65 years of age, the coordination of the specific immune response is disrupted because of the small number of naïve T cells associated with severe disease [176]. A protective immune response to infection is of the Th1 type, activated by the S epitopes and characterized by the synthesis of IFNγ, IL-2, and TNF-α, while a predominantly Th2 response, with IL-4, -5, -13 synthesis can have detrimental effects [107]. The intense response of follicular cytotoxic Th lymphocytes early in the disease was inversely correlated with the level of anti-protein S Ab [92]. Flow cytometry analysis of CD4+ T cells in patients and healthy donors stimulated with the S1 (C-terminal) and S2 (N-terminal) protein subunits show that most critically ill patients did not react to the N-terminal peptides of the S protein fragments containing RBD. Thus, the analysis indirectly suggests the critical protective role of CMI activated by the RBD sequence of protein S.

## 3. Conclusions

The evolution of SARS-CoV-2 infection is individual and is influenced by the peculiarities of immune reactivity. Eradication of the infectious process requires the activation of both compartments of the IR, innate and adaptive, humoral and cellular. IFN I synthesis is the primary, essential form of antiviral surveillance. IFN limits the multiplication of SARS-CoV-2, causing inapparent infection and mild form of the pathology. In the dysfunction of IFN synthesis, the virus multiplies in the lower respiratory tract, and the body’s self-defense mechanism activates the inflammatory reaction, during which the cascade of pro-inflammatory cytokine synthesis is activated. Excessive blood levels of pro-inflammatory cytokines cause multiorgan dysfunction. In the critical forms, the infection becomes viremic, as demonstrated by the presence of viral RNA in the plasma, forming platelet thrombi in the capillaries. The complex interaction between the dysregulated host immune response and COVID-19 severity enabled the rapid worldwide clinical evaluation of different immunomodulators, which are currently investigated in terms of their therapeutic effects in COVID-19 patients.

## Figures and Tables

**Table 1:** The mechanisms of the anti-infectious cutaneous-mucosal barrier.

Entry Gate	Local Defense Mechanisms	
Cutaneous tissue	Desiccation Acid pH Temperature variations Desquamation of epithelial cells Resident microbiota at this level (competition for adhesion and colonization sites) Hair follicles and sebaceous glands Subcutaneous lymphoid tissue	
Mucosa The mucin layer traps pathogens Submucosa lymphoid tissue	Conjunctival mucosa	The blinking reflex periodically removes pathogens that have reached this level Conjunctival secretion (sIgA)
	Nasopharynx	Resident microbiota Secretions (sIgA, phagocytes)
	Upper respiratory tract	Mucus Ciliary movements
	Lung	Macrophages
	Gastro-intestinal and genito-urinary tract	Periodic desquamation sIgA Complement Resident microbiota Low pH Proteolytic enzymes Low/High fluid velocity Low fluid velocity

**Table 2:** Effectors of innate and adaptive antiviral immunity.

Antiviral Immunity Components	Innate	Adaptive
Molecular factors	Intrinsic antiviral substances, interferons, complement, pro-inflammatory cytokines	Specific antibodies (IgG, IgM, IgA)
Cells	Natural killer (NK), phagocytes, dendritic cells (DCs)	T and B cells
Primary infection	+	+
Secondary Infection	+	+++
Immunological memory	−	+

**Table 3:** Proposed therapeutic strategies for reducing the deleterious effects of exacerbated inflammatory response in COVID-19 disease.

Drug Class	Target/Pathway	Name	Effect
Immunosuppressive/ Immunomodulatory drugs	Anti-inflammatory, anti-angiogenic, and anti-fibrotic immune modulator	Thalidomide	Combined with GC, prevents SARS-COV2 pneumonia [108]
	Anti-inflammatory	Lianhuaqingwen	Reduces TNF-α, IL-6, CXCL10, MCP-1 levels [109]
	Anti-inflammatory, prevents cellular autophagy	Chloroquine/ Hydroxychloroquine	Recovers the levels of NK cells and CD8+ T cells [110]
	Anti-inflammatory	Corticosteroids (dexamethasone)	Reduces death in severe cases [111] RECOVERY Collaborative Group, 2021
	Anti-inflammatory, immunosuppressive	Naproxen Indomethacin	Reduces viral load, suppresses RNA synthesis [112,113,114]
	Anti-inflammatory, antiviral	IFN-α2b	Reduces duration of detectable virus, reduces circulating levels IL-6, CRP [115]
		IFN-α2b with or without umifenovir	NCT04354259, NCT04343976, NCT04344600, NCT04388709 [116]
	Anti-inflammatory, NLRP3 inflammasome inhibitor	Rilonacept Inzomelid, Somalix, Dapansutrile (small molecule NLRP3 inhibitors)	[117,118,119]
	Activating and regulating immune cells Restores CD4+ and CD8+ T Cells Counts	Thymosin α1	Shorten viral RNA shedding duration and hospital stay [120,121]
	JAK signaling inhibitors	Baricitinib Fedratinib Ruxolitinib	Blockage of virus entry and the attenuation of host excessive inflammatory response NCT04970719 NCT04477993
	Serine protease inhibitors	Nafamostat mesylate Camostat mesylate	inhibitors of complement pathways and broad-spectrum anti-inflammatory agents [122]
	Modulation of the sphingosine-1-phosphate receptor 1 pathway	Fingolimod	sphingosine-1-phosphate receptor modulator, which sequesters lymphocytes in lymph nodes, preventing them from contributing to an autoimmune reaction NCT04280588 (https://clinicaltrials.gov/ct2/show/NCT04280588 (accessed on 5 December 2021)
Monoclonal antibodies	Antagonist of the IL-6 receptor	Tocilizumab and sarilumab	Reduce the cytokine storm [123,124]
	Recombinant mAb that binds to both soluble and membrane-bound IL-6	Siltuximab	NCT04330638
	Bruton’s tyrosine kinase (BTK) inhibitor- effects on the signaling of TLRs, IL-1R, CD19, BCR, CXCR4, and Fcγ-R1	Acalabrutinib Ibrutinib Acalabrutinib	[125] Clinical trials: NCT04375397 NCT04439006 NCT04564040 NCT04380688
	Recombinant antagonist of the IL-1 receptor	Anakinra, Gevokizumab, Canakinumab	Reduce death and hospitalization [119,126,127,128,129,130]
	Anti-TNF-α antibody	infliximab, adalimumab, certolizumab pegol	Reduce the cytokine storm [131,132] ChiCTR2000030089
	Anti-GM-CSF monoclonal antibody	Lenzilumab Gimsilumab Namilumab	Reduce the cytokine storm [133]
	Rhu GM-CSF	Sargramostim	[133,134,135,136]
	Partial opioid agonist	Meptazinol	Reduce the cytokine storm [137]
	MSC-based therapy	MSC-derived exosomes (MSC-Exo)	Trials ongoing [138,139,140]
Blood purification therapy	Hemodialysis, hemofiltration, plasma exchange, and hemoperfusion	Cytosorb	Reduce the cytokine storm [141,142]

Legend: AAK1-AP-2 associated protein kinase 1, GC—glucocorticoids, GM-CSF—granulocyte-macrophage colony-stimulating factor, SARS-CoV-2 main protease—CoV M^pro^, IFN—interferon, MSC—mesenchymal stem cell, Nab—neutralizing antibodies, Rhu GM-CSF—recombinant human granulocyte-macrophage colony-stimulating factor, TMPRSS2—transmembrane protease serine 2.

**Table 4:** Immunological markers used for the diagnosis and stratification of COVID-19 disease.

Parameter	Change	Specimen Type	COVID Status	Biosafety	Reference
Lymphocyte count	Lymphopenia	Blood	Increased severity	Clinical laboratory	[145]
Neutrophile count	Neutropenia	Blood	Increased severity	Clinical laboratory	[145]
ALT, AST, LDH, CRP, Ferritin	Elevated	serum	Increased severity	Clinical laboratory	[145]
IL-6	increased	serum	Critical illness	Clinical laboratory	[145]
D-dimer, lymphopenia	Elevated levels	serum	Risk of death	Clinical laboratory	[145]
IL-8	Moderately increased	serum	Moderate severity	Clinical laboratory	[146]
IFN γ	Not elevated	serum	All forms	Clinical laboratory	[146]
SARS-CoV 2 IgG antibody	Increased	serum	Chronic COVID Increased in positive older subjects	Clinical laboratory/biosafety level 2	[147,148]
SARS-CoV 2 IgM antibody	Increased	serum	Persistent COVID 10 symptoms and disease	Clinical Laboratory/biosafety level 2	[147,148]
Neutralizing antibodies	Increased	serum	Indicate immunity; monitor vaccine effectiveness	Clinical laboratory/biosafety level 3	[148]
